# Effect of bariatric surgery on nutritional and metabolic parameters: does the type of antidepressant medication matter?

**DOI:** 10.1007/s40519-024-01680-6

**Published:** 2024-07-25

**Authors:** Katherine J. P. Schwenger, Yasaman Ghorbani, Fadi Alkass, Tulasi Patel, Timothy D. Jackson, Allan Okrainec, Johane P. Allard

**Affiliations:** 1 Toronto General Hospital, University Health Network, Toronto, Canada; 2 Institute of Medical Science, University of Toronto, Toronto, Canada; 3 Department of Nutritional Sciences, University of Toronto, Toronto, Canada; 4 Division of General Surgery, University of Toronto, Toronto, Canada; 5 Division of General Surgery, Toronto Western Hospital, University Health Network, Toronto, Canada; 6 Division of Gastroenterology, Department of Medicine, Toronto General Hospital, University of Toronto, 585 University Avenue, 9N-973, Toronto, ON M5G 2N2 Canada

**Keywords:** Antidepressant, Selective serotonin reuptake inhibitor, Obesity, Bariatric surgery, BMI

## Abstract

**Purpose:**

Depression is prevalent in patients undergoing bariatric surgery (BSx). Long-term use of antidepressant is associated with weight gain, particularly the use of selective serotonin reuptake inhibitors (SSRIs). Little is known about whether different types of antidepressants affect the response to BSx. The purpose of this study was to determine the relationship between SSRI use and nutritional and biochemical measurements in those with obesity pre-/post-BSx.

**Methods:**

This is a cross-sectional and prospective cohort study. Patients were enrolled pre-BSx and divided into 3 groups: SSRI, non-SSRI and no antidepressant. Nutritional, biochemical and pharmacological data were collected pre- and 6 months post-BSx.

**Results:**

Pre-BSx, 77 patients were enrolled: 89.6% female, median age 45 years and body mass index (BMI) of 45.3 kg/m^2^. 14.3% were taking SSRIs and had a significantly higher BMI (52.1 kg/m^2^) compared to 62.3% in no antidepressant (46.0 kg/m^2^) and 23.4% in non-SSRI antidepressants (43.1 kg/m^2^). At 6 months post-BSx (*n* = 58), the SSRI group still had significantly higher BMI in comparison to the other two groups. No other significant differences found between groups.

**Conclusion:**

Despite higher BMI, patients taking SSRI and undergoing BSx had similar responses, based on nutritional and biochemical parameters, to those on non-SSRI or no antidepressants.

**Level of evidence:**

Level III: Evidence obtained from well-designed cohort or case–control analytic studies.

## Introduction

Clinical depression has a lifetime prevalence reported to be up to 40% in individuals living with obesity, including patients undergoing bariatric surgery (BSx) [1]. Obesity and depression illustrate a bidirectional relationship, as obesity can lead to a higher risk of depression and taking anti-depressants can induce weight gain [1, 2]. Factors such as genetics, binge and emotional eating, poor sleep and poor nutrition can further influence the bidirectional relationship [2].

Various treatment regimens are used to treat depression including psychotherapy and antidepressants such as selective serotonin reuptake inhibitors (SSRI), serotonin-norepinephrine reuptake inhibitors (SNRI), tricyclic antidepressants (TCA), monoamine oxidase inhibitors (MAOI) and other agents [3]. These antidepressants work through various mechanisms, resulting in patients experimenting with several different treatment regimens before finding the right medication(s) [3]. Among the many classes of antidepressants, SSRIs are the most prescribed medication as the first line of treatment for depression [4, 5]. However, paradoxically, SSRIs can also contribute to the underlying cause of depression, which can be obesity, by inducing further weight gain. However, the metabolic mechanism linking the biology of weight gain in association with psychotropic medication is poorly understood but murine models suggest a change in activity of orexigenic regions in the brain [6].

Only one study looked at the types of antidepressants pre- and post-BSx. However, they only assessed the excess BMI loss while other metabolic and nutritional parameters were not assessed [7]. Therefore, this study aims to investigate the relationship between the types of antidepressants used and the effect of BSx by assessing nutritional and biochemical measurements pre- and 6 months post-BSx.

## Materials and methods

In this cross-sectional and then, prospective cohort study, subjects were recruited from the bariatric program at University Hospital between December 2015 and August 2021. Inclusion criteria consisted of age ≥ 18 years and being approved for BSx following the Canadian Bariatric Surgery guidelines [1]. For those who decided to undergo BSx and met further study criteria for the cohort study, nutritional and biochemical parameters were re-assessed at 6 months. The cohort study criteria were based on a concomitant larger microbiome study and excluded patients with previous surgery involving gastrointestinal tract modification, type 1 diabetes, pregnancy, smoking, and regular use of prebiotics, probiotics, antibiotics, and non-steroidal anti-inflammatory drugs within three months of the study participation. Upon informed consent, subjects’ clinical data (nutritional, biochemical and medication assessments) were collected at baseline and 6 months post-BSx.

### Nutritional assessment

Subjects’ height and weight were measured at baseline and 6 months post-BSx to calculate BMI (kg/m^2^). Percent excess body weight loss (% EBWL) was calculated for each subject 6 months post-BSx using (% EBWL = 100 × [weight loss since BSx /preoperative excess body weight]). In addition, each subject was assigned a three-day food log to record all meals consumed in 24-h periods, including one weekend. Portion sizes were estimated by applying the 2D Food Portion Visual Chart (Nutrition Consulting Enterprises, Framingham, MA). Data were reviewed and entered by an RD into Food Processor Diet and Nutrition Analysis Software (Version 7, ESHA Research, Salem, OR).

### Biochemical

Fasting plasma and serum samples were collected at baseline and 6 months post-BSx to measure glucose, insulin, hemoglobin A1c (HbA1c), and c-peptide, which was analyzed via Hospitals Laboratory Medicine Program using standard laboratory tests. The fasting glucose and insulin were used to calculate the homeostasis model assessment for insulin resistance (HOMA-IR). Subjects who scored ≥ 2.73 were considered insulin-resistant.

### Antidepressant

A complete medication list was collected during both baseline and 6 months post-BSx; antidepressants were categorized according to the antidepressant classes: SSRI, SNRI, TCA and atypical antidepressants. Subjects were organized into the following groups: SSRI, non-SSRI (SNRI, TCA, and atypical antidepressant users) or no-antidepressant groups.

### Statistical analysis

All data and any statistical calculations were performed using the SAS 9.4 program (SAS Institute, Cary, NC, USA). Various tests such as paired t-test, Kruskal–Wallis test, Wilcoxon ranked sum, or χ^2^ and Fisher’s exact test were used to compare parameters of interest between groups. Statistical significance was considered when *p* < 0.05.

## Results

A total of 77 subjects were included in the cross-sectional study, and 58 participated in the 6-month prospective study. Subjects’ characteristics are presented in Table 1. At baseline, 69 (89.6%) subjects were female, and the median age was 45 (38, 52 [1st quartile, 3rd quartile]) years with a BMI of 45.3 (42.5, 50.7) kg/m^2^ and waist circumference of 131 (124, 139) cm. For those completing the 6-month post-BSx follow-up and regardless of the antidepressant use, BMI and waist circumference decreased significantly (Table 1). Dietary intake 6 months post-BSx significantly changed. Specifically, total energy and carbohydrate intake (as a percentage of energy intake) significantly decreased, while protein intake significantly increased (as a percentage of energy intake).

**Table 1:** Nutritional intake, biochemical and anthropometric characteristics at baseline and 6 months post-bariatric surgery

Variables	Baseline (*n* = 77)	6 months post-bariatric surgery (*n* = 58)	*p*-value
Age (years)	45 (38, 52)	–	–
Sex *n* (% female)	69 (89.6)	50 (86.2)	–
Waist (cm)	131 (124, 139)	103 (98, 110)	**< 0.0001**
BMI (kg/m^2^)	45.3 (42.5, 50.7)	34.2 (31.7, 37.6)	**< 0.0001**
Glucose (mmol/L)	5.3 (4.8, 6.0)	4.7 (4.4, 5.1)	**< 0.0001**
Insulin (pmol/L)	132 (93, 177)	53.0 (36.0, 63.0)	**< 0.0001**
HOMA-IR	5.2 (3.7, 8.1)	1.9 (1.2, 2.2)	**< 0.0001**
HbA1c	0.06 (0.05, 0.06)	0.05 (0.05, 0.06)	**< 0.0001**
C-peptide (pmol/L)	1202 (947, 1513)	655 (367, 828)	**< 0.0001**
Total energy intake (kcal)	1713 (1387, 2290)	1066 (841, 1294)	**< 0.0001**
Fat (% energy intake)	35.7 (29.9, 43.1)	38.0 (32.6, 45.2)	0.4373
Carbohydrate (% energy intake)	44.9 (38.6, 52.6)	38.3 (29.5, 44.8)	**0.0277**
Protein (% energy intake)	17.4 (14.7, 21.4)	24.3 (18.6, 32.4)	**0.0112**

Data presented as median (1st quartile, 3rd quartile) or *n* (%) as appropriate. Significant values are shown in bold

*BMI* body mass index, *HbA1c* hemoglobin A1C, *HOMA-IR* homeostatic model of assessment for insulin resistance

The patients were then classified into three categories based on antidepressant use at baseline: patients taking SSRIs (*n* = 11), non-SSRI antidepressants (*n* = 18) and no-antidepressant group (*n* = 48). These categories were compared at baseline (see Table 2) and at 6 months post-BSx (see Table 2). At baseline, those on SSRIs had a significantly larger waist circumference than the non-SSRI antidepressants group and a significantly larger BMI compared to the other two groups. At 6 months post-BSx, all 3 groups showed a reduction in BMI and waist circumference but those on SSRIs had a significantly higher BMI at 6 months post-BSx than no-antidepressant group.

**Table 2:** Baseline nutritional intake, clinical and biochemical variables between patients taking SSRI and those not taking antidepressants, and SSRI and those taking other antidepressants

	Baseline			6 months post-bariatric surgery			Change between baseline and 6 months post-bariatric surgery		
	No anti-depressant (n = 48)	Non-SSRI anti-depressants (n = 18)	SSRI (n = 11)	No anti-depressant (n = 40)	Non-SSRI anti-depressants (n = 8)	SSRI (n = 10)	No anti-depressant (n = 40)	Non-SSRI anti-depressants (n = 8)	SSRI (n = 10)
Age (years)	46 (38, 52)	48 (38, 51)	42 (36, 61)	–	–	–	–	–	–
Waist circumference (cm)	132 (126, 137) ^a^	123 (121, 131) ^a, b^	141 (131, 149)^b^	103 (98, 110)	103 (99, 109)	112 (103, 125)	− 28 (− 32, − 20)	− 21.8 (− 30, − 15)	− 25 (− 35, − 17)
BMI (kg/m^2^)	46.0 (42.9, 49.2) ^a^	43.1 (41.0, 47.4) ^b^	52.1 (44.4, 58.1)^a, **b**^	33.8 (31.8, 37.3)^c^	33.4 (30.6, 36.22)	40.4 (34.7, 45.4)^c^	− 11.4 (− 14.8, − 9.7)	− 12.2 (− 13.7, − 7.5)	− 12.9 (− 15.9, − 10.8)
%EBWL	–	–	–	–	–	–	40.5 (34.6, 46.5)	40.7 (29.9, 43.1)	36.5 (28.3, 41.5)
Glucose (mmol/L)	5.3 (4.8, 6.1)	5.4 (5.1, 6.0)	5.5 (5.1, 7.0)	4.7 (4.4, 5.1)	4.7 (4.3, 5.2)	4.9 (4.3, 5.2)	− 0.75 (− 1.25, − 0.2)	− 0.6 (− 1.0, 0.5)	− 0.6 (− 1.1, − 0.2)
Insulin (pmol/L)	142 (95, 195)	131(93, 151)	123 (63, 147)	50 (33.50, 65.00)	55 (52, 58)	52 (37, 62)	− 52 (− 87, − 18)^a^	− 97 (− 120, − 77)^a^	− 69 (− 78, − 55)
HOMA-IR	5.3 (3.7, 8.9)	5.1 (4.1, 6.7)	5.2 (2.2, 7.0)	1.8 (1.2, 2.2)	1.8 (1.7, 1.9)	1.6 (1.0, 2.4)	− 2.6 (− 4.8, − 1.0)	− 3.4 (− 4.0, − 3.2)	− 2.5 (− 3.2, − 2.1)
HbA1c	0.06 (0.05, 0.06)	0.06 (0.06, 0.07)	0.06 (0.05, 0.07)	0.05(0.05, 0.05)	0.05 (0.05, 0.06)	0.05 (0.05, 0.06)	0.00 (− 0.01, 0.00)	− 0.00 (− 0.01, − 0.00)	− 0.01 (− 0.01, 0.00)
C-peptide (pmol/L)	1284 (834, 1615)	1104 (990, 1307)	957 (798, 1205)	621 (361, 890)	700 (669, 844)	589 (462, 688)	− 489 (− 838, 259)	− 589.00 (− 861, − 552)	− 741 (− 1105, − 368)
Total energy intake (kcal)	1605 (1375, 2237)	1860 (1483, 2378)	1889 (1460, 2666)	1063 (964, 1309)	1260 (1190, 1587)^a^	699 (642, 1110)^a^	− 421 (− 1054, − 81)	− 537 (− 633, − 344)	− 824 (− 1680, − 766)
Fat (% energy intake)	33 (30, 42)	39 (35, 43)	39 (35, 45)	40 (34, 45)	40 (31, 51)	34 (32, 38)	4.1 (− 5.2, 8.5)	− 3.9 (− 10.8, 7.2)	5.0 (− 4.0, 5.1)
Carbohydrate (% energy intake)	47 (40, 53)	43 (36, 47)	41 (38, 48)	38 (33, 45)	35 (30, 44)	38 (25, 45)	− 4.3 (− 13.9, 2.6)	− 4.7 (− 13.5, 1.7)	− 14.9 (− 15.4, − 9.6)
Protein (% energy intake)	17 (15, 21)	18.4 (14, 23)	18 (14, 23)	22 (18, 25)^a^	22 (17, 30)	35 (30, 35)^a^	1.2 (− 2.9, 7.2)^b^	3.9 (0.6, 15.2)	15.6 (11.4, 22.2)^b^

Data presented as median (1st quartile, 3rd quartile). Values with identical superscript are significantly different (*p* < 0.05)

*BMI* body mass index, *%EBWL* % excess body weight loss, *HbA1c* hemoglobin A1C, *HOMA-IR* homeostatic model of assessment for insulin resistance

For dietary composition, baseline parameters were comparable between the three groups. However, when comparing their 6 months dietary composition, after BSx, those on SSRIs consumed significantly higher protein (as percentage of energy intake) compared to those not taking any other antidepressants. There was no significant difference between groups in terms of percentage changes in other parameters (data not shown).

## Discussion

In the cross-sectional study, before undergoing BSx, patients taking SSRI had greater BMI and waist circumference compared to those taking non-SSRI antidepressants and those not taking any antidepressants. The overall effect of BSx on biochemical and nutritional measurements was similar between the three groups, but the SSRI group tended to remain with higher BMI.

Our study population is comparable to other reports, in terms of age, sex and BMI [1]. Similar to another study [4], more than a third (37.7%) of our subjects were prescribed antidepressants prior to BSx. One study reported that more than half their candidates were on SSRI antidepressants [4] while 37.9% of our patients using anti-depressants took SSRI, suggesting that this type of anti-depressant is frequently prescribed and an effective treatment.

In the cross-sectional study, the SSRI group tended to have more severe obesity based on BMI and waist circumference, but dietary intakes and biochemical parameters were similar to the other groups. Possible mechanisms for the anthropometric difference may relate to lower exercise level in the SSRI group or genetics (e.g., polymorphism) which could increase the risk of side effects from antidepressants [8]. This was not assessed during this study. Changes in activity of the feeding inhibition system in the hypothalamus has also been reported [6] but we did not detect differences in dietary intakes in the cross-sectional study. Others have reported different dietary intakes. The National Health and Nutrition Examination Survey found that individuals taking antidepressants, although having a similar diet composition, had a higher total energy intake associated with higher BMI compared to those not taking antidepressants [5]. However, the type of anti-depressant was not specified. In contrast, two other studies comparing bariatric subjects with antidepressants versus without found no significant difference in their pre-BSx weights [9, 10].

To the best of our knowledge, there are no other studies on SSRI and weight in bariatric subjects but there are several studies in the non-obese population which showed associations between the use of SSRI and increased BMI [11–13]. One study in those with panic disorder using SSRI found that there is an average of 5–8 kg weight gain after 12 months [11]. Another study in individuals diagnosed with major depressive disorder found that one-year treatment with fluoxetine, an SSRI, resulted in 3.1 kg weight gain [12]. Additionally, in drug-naïve subjects with anxiety, 16-week treatment with either fluoxetine or paroxetine (another SSRI) resulted in a significant increase in BMI and waist circumference but such effect was not seen in other SSRIs (i.e., citalopram, sertraline and escitalopram) [13].

Based on this literature, we wanted to determine if the use of SSRI could affect the response to BSx and found that, overall, the subjects on SSRI had a similar response to those on non-SSRI or no antidepressant medication but the SSRI group tended to remain with higher BMI 6 months post-BSx. However, other studies with similar patient demographics and BMI range found no differences between no-antidepressant and antidepressant groups at 6 months post-BSx but specific classes of antidepressants were not assessed [9, 10]. This is relevant since the use of SSRI may be disadvantageous to the weight loss post-RYGB.

Another way to assess response to BSx is to look at %EBWL. We found no significant difference in %EBWL between SSRI, non-SSRI and no-antidepressant groups at 6 months post-BSx. Similar to our study, Love et al. [9] compared those on antidepressants and those on no antidepressants and found no significant difference in their percentage of weight loss at either 6 months or 1 year post-BSx. However, another study using percentage of excess BMI loss instead of %EBWL found that continuing antidepressants in the first-year post-BSx was associated with a significantly lower percentage of excess BMI loss [7]. One reason for their different findings could be that their population had a lower pre-BSx BMI of 41.95 kg/m^2^ [7] compared to 45.3 kg/m^2^ in our population. In the same study, using a multivariate analysis investigating the percentage of excess BMI loss, the authors found that overall antidepressant use (as well as SNRI and TCA) significantly reduced the percentage of excess BMI loss compared to those on no antidepressants. They did not find such relationship for the SSRI group [7]. Waist circumference was not assessed in that study [7].

Our results also showed that those taking SSRIs still had a BMI category of Obese Class III (40.4 kg/m^2^), whereas those not taking any antidepressants (33.8 kg/m^2^) and those taking non-SSRI antidepressants (33.4 kg/m^2^) had a BMI category of Obese Class I 6 months post-BSx [14]. Overall, in our study, the amount of weight loss was comparable between all three groups, indicating that SSRI use does not impede weight loss but is associated with higher weight before the BSx, thus making it more challenging to achieve better target weight/BMI post-BSx.

Despite finding that the SSRI group tended to be more obese than the other groups at 6 months post-BSx, their dietary assessments showed less total energy intake, but significantly higher protein intake compared to those on non-SSRI antidepressants and those on no antidepressants, respectively. There are no specific explanations for this. However, given the role of depression in poor dietary choices [15], it is possible that treatment with SSRI in conjunction with BSx and dietary counselling favored lower energy and higher protein intakes in this group compare to the other groups. Also, higher protein intake is known to curb appetite [16] and may have had an effect in reducing total energy intake. These results suggest a need for further studies on the influence of SSRI in dietary intakes, especially post-BSx. However, despite these differences, there were no other significant differences between nutritional, anthropometric and biochemical data between the three groups.

## Strength and limits

The strengths of this study include the use of a homogenous bariatric population, its prospective nature and the analysis of the dietary intake as a potential contributing factor to the differences in BMI between those taking SSRIs and those not taking any antidepressants. There are a few limitations. This was a single-center study, which limits generalizability, and the sample size was small compared to similar patient populations in previous studies [1, 4]. Similar to other studies [7, 9, 10], we did not consider different types of SSRIs depending on their mechanism. However, we collected more nutrition and laboratory data that extended our knowledge about the relationship between antidepressants, dietary intake, and nutritional status in bariatric patients. This study was part of a larger study protocol that did not include questionnaires assessing depression or the type of depression, but prescribed antidepressants were recorded.

## Conclusions

In conclusion, while the BMI was significantly higher pre-BSx in those taking SSRIs compared to non-SSRI and no-antidepressant groups, the overall change in BMI and % EBWL were comparable between groups 6-month post-surgery. However, those on SSRI remained in Obesity Class III with higher BMI versus those without antidepressants. Therefore, prescribing SSRIs in bariatric patients may create a disadvantage in regards to BMI overall and to post-BSx target weight and BMI. However, considering the study design and sample size, our results should be interpreted with caution. Future randomized trials with larger sample size may be considered to investigate the relationship between specific classes of antidepressants, especially SSRIs, and their effects on BMI as well as on food intake and food choices.

### What is already known on this subject?

Many people who have obesity or undergo bariatric surgery have clinical depression and take antidepressants. However, antidepressants can have a bidirectional relationship with obesity. The relationship between the antidepressants and post-bariatric surgery nutritional and biochemical outcomes remains poorly understood.

### What this study adds?

Patients taking SSRI had significantly higher BMI at baseline and 6 months post-bariatric surgery compared to those on non-SSRI or without antidepressants. To observe the full benefits of weight loss via bariatric surgery in patients with depression, patients may need to take non-SSRI antidepressants.

## Data Availability

The datasets generated during and/or analyzed during the current study are available from the corresponding author on reasonable request.

## Abbreviations

BMI: Body mass index
%EBWL: Percent excess body weight loss
HbA1c: Hemoglobin A1c
HOMA-IR: Homeostatic model of assessment for insulin resistance
MAOI: Monoamine oxidase inhibitors
RD: Registered dietitian
SNRI: Serotonin-norepinephrine reuptake inhibitors
SSRI: Selective serotonin reuptake inhibitor
TCA: Tricyclic antidepressants
MAOI: Monoamine oxidase inhibitors
BSx: Bariatric surgery
